# The prevalence of human papillomavirus in ocular surface squamous neoplasia in HIV positive and negative patients in a South African population

**DOI:** 10.1007/s15010-024-02289-8

**Published:** 2024-05-09

**Authors:** Loren N. Odendaal, Corinna Andreae, Micheline Sanderson-November, Dan Zaharie, Derrick P. Smit

**Affiliations:** 1 Division of Ophthalmology, Faculty of Medicine and Health Sciences, Stellenbosch University, Room 5053, Clinical Building, Francie Van Zijl Drive, Parow Valley, 7505 South Africa; 2 Department of Anatomical Pathology, Faculty of Medicine and Health Sciences, Stellenbosch University, Francie Van Zijl Drive, Parow Valley, 7505 South Africa

**Keywords:** Human papillomavirus, Human immunodeficiency virus, Ocular surface squamous neoplasia

## Abstract

**Purpose:**

To assess the prevalence and subtypes of Human Papillomavirus (HPV) in Ocular Surface Squamous Neoplasia (OSSN) in Human Immunodeficiency Virus (HIV) positive and negative patients in South Africa.

**Basic procedures:**

This study was a single center retrospective cross-sectional study, conducted at Tygerberg Hospital, Western Cape, South Africa. We assessed 63 histopathologically confirmed OSSN formalin-fixed paraffin-embedded (FFPE) tissue blocks from 2015–2023. The presence of HPV was determined using the Hybrispot Direct Flow Chip Kit. Corresponding clinical data was retrieved from the National Health Laboratory Service (NHLS) central data warehouse.

**Main findings:**

Of the confirmed OSSN samples, 66.7% tested positive for HPV (95% confidence interval [CI] 54–77.3%). Of the 42 HPV positive samples, 38 (90.5%) had one or more known genotypes detected and 4 had unknown genotypes. The most prevalent subtypes were HPV 11, 16 and 18 (found in 61.9%, 52.4% and 33.3% of HPV positive samples respectively). 88.9% of the lesions biopsied were from HIV positive patients, of whom 56.4% had a CD4 + count of < 200 cells/µL. A lower median CD4 + count was detected among HIV positive patients with invasive squamous cell carcinoma compared to those with moderate dysplasia (p < 0.0198).

**Conclusions:**

There is a high prevalence of HPV in OSSN in South Africa. Certain subtypes namely, 11, 16, 18, 31, 33 and 35 may be more carcinogenic. HIV with HPV co-infection may be linked as a causative factor in the development of OSSN.

## Introduction

Ocular surface squamous neoplasia (OSSN) is an umbrella term that describes a spectrum of pre-malignant and malignant epithelial lesions of the conjunctiva and cornea [1–4]. It encompasses a continuum of histological findings, namely dysplasia, carcinoma in situ and squamous cell carcinoma of the conjunctival and/ or corneal epithelium. [1–3] There are a few known pathogenetic factors, for example UV radiation, but in the last two decades it has been speculated that Human Papillomavirus (HPV) could play a role in OSSN development. A review in 2019 by Chalkia et al. noted the prevalence of HPV in OSSN to vary widely from 0–100%, with an average prevalence of 33.8%. This review found that the HIV status of majority of these patients was unknown and that the discrepancies between the prevalence in different studies render the role of HPV in OSSN uncertain [5]. Most African studies have found that 75–85% of OSSN lesions are positive for HPV with varying subtypes. [6–8] In high income countries, OSSN is mainly seen in elderly Caucasian patients, but in low-middle income countries such as South Africa it is noted mainly in young HIV positive patients [9–13]. This prompted further investigation into the prevalence of HPV in OSSN lesions of HIV positive and negative patients in South Africa. To our knowledge this is the first such study to report on HPV prevalence in OSSN lesions in a South African population. Further understanding of the pathogenetic mechanisms and subtypes of HPV in OSSN may be of significance to future preventative measures, such as HPV vaccines [13].

## Methods

### Study design and ethics

This study was a single center retrospective cross-sectional study, conducted at Tygerberg Hospital, Western Cape, South Africa. Ethics approval was obtained from the Stellenbosch University Health Research Ethics Committee (05 November 2021, Reference No. S20/06/137). A waiver of consent was obtained for all stored tissue samples and clinical information collected. The study was performed according to the ethics of the Declaration of Helsinki. Methodology followed recommendations of the STROBE statement for reporting cross-sectional studies.

### Outcomes and sample selection

Our primary outcome was to establish the prevalence of HPV in OSSN in our setting. Secondary outcomes consisted of determining specific HPV subtypes in OSSN, establishing correlation with HIV status, and other demographic factors in OSSN. A central NHLS database and theatre archive search from 2015–2023 revealed potential samples of OSSN stored in the Anatomical Pathology archive. A database was populated with the available data after all participants were anonymized. Variables collected included age at time of biopsy, biological sex, HIV status, viral load (VL) and CD4 count. The VL and CD4 count closest to the time of biopsy were used. There was no more than one sample per patient in this study. Inclusion criteria were that participants should be 18 years and older at time of tissue biopsy and that samples were histologically confirmed to be OSSN. Exclusion criteria were any samples damaged during biopsy with an unclear diagnosis or samples that were insufficient for Hybrispot testing.

### Tissue processing

A pathologist (DZ) confirmed the histopathological diagnosis of OSSN of each sample. Two to four 10 µm sections, depending on the tissue size, were used for DNA extraction. The first two tissue sections were discarded to remove exposed tissue surface, to reduce exposure to nucleases. A new microtome blade was used to cut each formalin-fixed paraffin-embedded tissue (FFPE) tissue block, and the microtome was cleaned using 10% (v/v) bleach and 70% (v/v) ethanol after sectioning of each FFPE block. Paraffin was removed from tissue sections using the deparaffinization solution (catalogue #19,093) (Qiagen, Hilden, Germany). Total DNA was extracted using the QIAamp DNA FFPE Tissue Kit (Qiagen, Hilden, Germany) according to manufacturer’s recommendations. The quality of the extracted DNA was assessed by measuring the A260_nm_/A280_nm_ and A260_nm_/A230_nm_ absorbance ratios using the Biodrop µLite spectrophotometer (Biodrop LTD, Cambridge, UK). DNA concentrations were determined using the Qubit 3.0 fluorometer and the Qubit dsDNA BR assay (catalogue# Q328500) (Life Technologies, Thermo Fisher Scientific, California, USA) according to manufacturer’s recommendations. Additionally, DNA integrity was evaluated through the Polymerase chain reaction (PCR) amplification of a 205 bp fragment of the human β-globin gene using a SYBR green based quantitative Real-Time Polymerase Chain Reaction (qRT-PCR) adapted from the method of (Greer et al., 1990). DNA was stored at − 20 °C until further analysis.

### HPV genotype determination

A minimum of 100 ng of extracted DNA was used for HPV genotyping using the HybriSpot Direct Flow Chip kit (Master Diagnóstica, Granada, Spain) according to the manufacturer’s instructions. Detection and genotyping of HPV viral DNA by PCR and reverse dot blot hybridization was performed. Automatic analysis of results reported on presence of high-risk genotypes: 16, 18, 26, 31, 33, 35, 39, 45, 51, 52, 53, 56, 58, 59, 66, 68, 73, 82, and low-risk genotypes: 6, 11, 40, 42, 43, 44/55, 54, 61, 62/81, 67, 69, 70, 71, 72, 84. High risk and low risk subtypes were specified as such by the testing kit and were based on an epidemiological classification of HPV subtypes associated with cervical cancer by Muñoz in 2003. [14]

### Data Analysis

Data captured into the study database was exported into Stata 18 for analysis. Descriptive statistics were used to describe individuals identified from 2015–2023 with OSSN and tested for presence of HPV genotypes in the lesions. Appropriate tables and graphs were used to visualise the data. Analysis methods for the primary and secondary objectives are described below:

- The prevalence of HPV in all biopsied OSSN lesions seen in HIV positive and negative patients presenting to the Division of Ophthalmology at Tygerberg Hospital from 2015–2023 was calculated and presented as a percentage with a 95% confidence interval.
- Descriptive statistics were used for the demographic and clinical characteristics of patients with OSSN and tested for HPV. Median and interquartile ranges were used for continuous variables while frequencies and percentages were used for categorical data. The patients were described in terms of age, biological sex, HIV status, most recent CD4 count and viral load to the time of OSSN diagnosis.
- The prevalence of specific HPV genotypes in the OSSN specimens was determined as the proportion of specimens that tested positive for any of the HPV genotypes included in the assay.

## Results

### HPV prevalence

Of the 63 OSSN lesions identified, 1 (1.6%) had mild dysplasia, 5 (7.9%) had moderate dysplasia, 33 (53.4%) were identified as squamous cell carcinoma in-situ (SCCIS) and 24 (38.1%) were found to be invasive squamous cell carcinomas (ISCC). The proportion that tested HPV positive with Hybrispot testing was 42/63 (66.7%) (95% confidence interval [CI] 54–77.3%). Table 1 outlines the demographic and clinical characteristics of the 63 patients with OSSN who were tested for HPV.

**Table 1:** The demographic and clinical characteristics of patients with OSSN lesions and tested for HPV, N = 63

Characteristic	n (%)
Age in years (median, IQR)	42 (35- 47)
Biological sex:	
Female	38 (60.3)
Male	25 (39.7)
HIV status:	
Negative	3 (4.8)
Positive	56 (88.9)
Unknown	4 (6.4)
CD4 count (median, IQR)*	157 (113–369)
CD4 counts < 200 cells/µl*	31 (56.4)
VL categories (copies/ml)**	
< 50	26 (46.4)
50–1000	13 (23.2)
> 1000	17 (30.4)

*Available for 55/56 HIV positive patients with OSSN lesions and tested for HPV

**Available for 54 /56 HIV positive patients with OSSN and tested for HPV

#### Genotypes in HPV positive samples

**Fig. 1:**
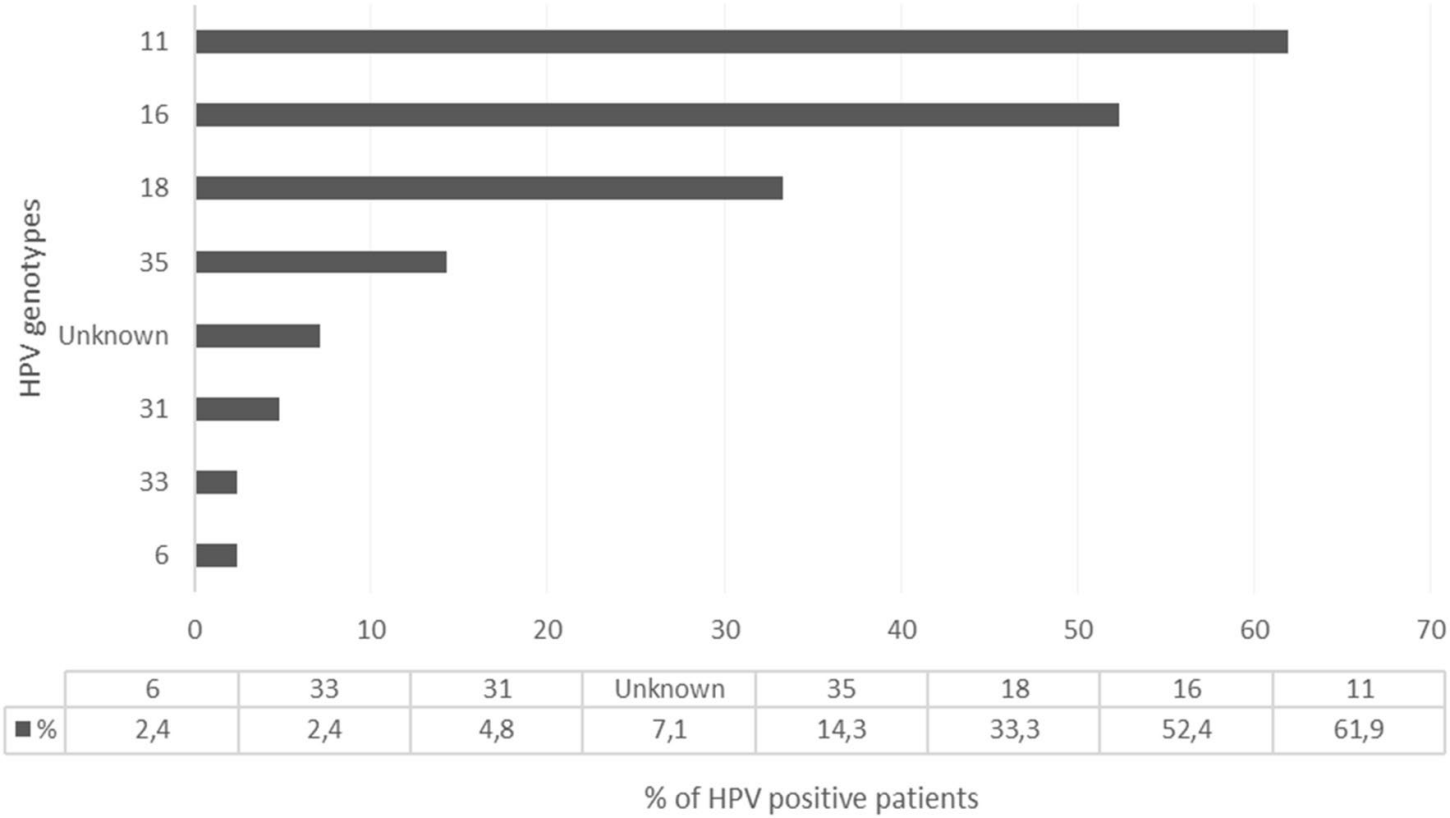
HPV genotypes identified in the HPV positive samples

Of the 42 HPV positive samples, 38 (90.5%) had one or more known genotypes detected and four (9.5%) had unknown genotypes. Figure 1 represents the HPV genotypes identified in the HPV positive samples. The most common genotype in mono-infected samples was HPV 11 (8 samples), HPV 16 (7 samples) and HPV 18 (2 samples) followed by HPV 6 and 35 (each found in 1 sample only). HPV subtypes 31, 33 and 52 were never identified in isolation and were most commonly co-infected with HPV 11, 16 or 18. The most common genotypes in multiple co-infected samples was a combination of HPV 11, 16 and 18 respectively. Among those with known genotypes detected, the number of genotypes were distributed as follows:

- 21 (55.3%) patients had 1 genotype,
- 6 (15.8%) had 2 genotypes,
- 6 (15.8%) had 3 genotypes,
- 4 (10.5%) had 4 genotypes and
- 1 (2.6%) had 5 genotypes.

#### Viral Load and CD4 count distribution

OSSN sample histological grading and HIV viral loads were compared. In the moderate dysplasia, in situ and invasive squamous cell carcinoma groups, the number of patients with VL > 1000/ml were 2, 8 and 7 respectively. There was no statistically significant correlation between VL and OSSN grade. (p < 0.790).

The median CD4 count for all HIV positive patients with OSSN was 157 cells/µl (IQR 113- 369). CD4 counts by OSSN grade were as follows:

- Moderate dysplasia: 458 (IQR 243—701)
- SCCIS: 152 (IQR 91.5- 375)
- ISCC 160 (IQR 113 – 217)

(K-test for equality of medians p value = 0.308).

**Fig. 2:**
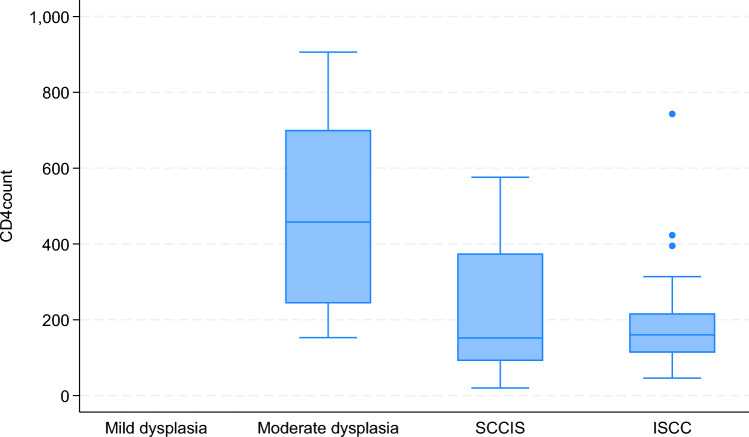
CD4 count of each patient compared to their OSSN histological grading

Figure 2 compares the CD4 count of each patient with OSSN to the histological grading of dysplasia present in the lesion. There was a statistically significant difference between median CD4 counts among HIV positive patients with moderate dysplasia to those with invasive SCC (p value for Rank sum test = 0.0198).

## Discussion

The worldwide prevalence of HPV in OSSN is about 33.8%, with a much higher prevalence in African countries of 75–85%, however, many previous studies did not report on the HIV prevalence [5]. Our HPV prevalence of 66.7% in OSSN is more in keeping with that of other low-middle income countries where there is also a higher percentage of HIV positive patients in the population. In comparison, in a high-income country such as Germany, a study in 2009 reported a 0% HPV prevalence in 31 OSSN samples, but all these samples were from HIV negative patients. The median age of their 26 male and 5 female Caucasian patients was 70 (range 42–91) years, which is a lot older than our median age of 42 [15].

The most common subtypes of HPV found in OSSN in various countries, as noted by a review by Chalkia et al., are 6,11,16 and 18 [5]. We used the Hybrispot testing method which tests for known high risk and low risk subtypes. While subtypes 6 and 11 identified in our samples are low risk, the subtypes 16, 18, 31, 33 and 35 identified in our samples are known to be high risk for their carcinogenic potential. Our study suggests that more than one genotype could simultaneously be involved in the pathogenesis of OSSN. HPV subtype 11 was most prevalent in this cohort, and in 8 of the OSSN samples it was the only HPV subtype identified. HPV 11 is thought to have a low carcinogenic potential in cervix carcinoma, but further studies would be required to assess its role in OSSN. Quantitative studies would be needed to assess whether HPV is actively replicating and therefore possibly pathogenic in the malignancy or if the virus is simply latent in the lesion. We had 4 samples that tested positive for HPV but where the subtype could not be determined. The Hybrispot testing kit stipulates that in these samples the specific genotype is not included in the testing panel. There is, therefore, a possible subtype involved in the pathogenesis that is not previously known to be carcinogenic. Further testing such as Next Generation Sequencing, although not done in this study, may be able to identify these subtypes.

HIV is known to be a risk factor for OSSN. In the African continent the prevalence of HIV in OSSN is particularly high, with Zimbabwe having found a prevalence of 91% [13]. A recent study at a tertiary institution in Gauteng, South Africa, reported a prevalence of HIV of 74% in patients with OSSN (p < 0.001) [16]. This is in keeping with the finding in our study of 88.9% HIV prevalence in patients with OSSN. In our study, 2 HIV negative patients with OSSN tested positive for HPV. The subtype found in the first sample was HPV 16, and in the second sample HPV 11 and 35. The possible limitation to the accuracy of our HIV prevalence include the window period of HIV and patients having an unknown status.

According to an article studying the epidemiology of OSSN in Africa, no linear association between CD4 count and OSSN has been established [17]. Our results show a statistically significant difference between median CD4 counts among HIV patients with moderate vs invasive SCC (p < 0.0198). This could suggest that the lower the CD4 count of the patient, the more invasive the OSSN. Further studies would be needed to assess this correlation as our sample size of invasive SCC was much smaller than those with moderate dysplasia. No significant correlation between HIV viral load and OSSN grade was noted.

A key question for future research would be whether more comprehensive HIV treatment and control and HPV vaccination could reduce the burden of HIV associated malignancies. Due to the retrospective nature of the study, and limited clinical records available, the HPV vaccination status and HIV treatment profile of this study population was unknown. Three HPV vaccines namely; 9-valent HPV vaccine (Gardasil 9, 9vHPV, Merck & Co. Inc., Rahway, NJ, USA), quadrivalent HPV vaccine (Gardasil, 4vHPV, Merck & Co. Inc., Rahway, NJ, USA) and bivalent HPV vaccine (Cervarix, 2vHPV, GlaxoSmithKline, London, UK), have been licensed by the U.S. Food and Drug Administration (FDA). 2vHPV protects against HPV 16 and 18, whereas 4vHPV provides protection against HPV 6,11,16 and 18 and 9vHPV protects against HPV 6,11,16,18,31,33,45,52 and 58 [18]. Only 31% of African countries have implemented the HPV vaccination as part of their national immunisation programmes, compared with 77% and 85% of countries in Europe and the Americas, respectively [19]. Further identification of potentially carcinogenic HPV subtypes could aid in directing HPV vaccines against subtypes that are implicated in a particular population group. The HPV vaccine was approved by the South African Health Products Regulatory Authority in 2008 and in 2014 the government introduced free vaccination against HPV to girls in public schools [20]. The vaccine currently used in South Africa is the bivalent vaccine which, therefore, only covers HPV 16 and 18. An HPV vaccine effective against multiple strains could provide better protection against HPV associated cancers, thereby possibly reducing the burden not only of cervical cancer but other cancers such as OSSN.

## Conclusion

This is the first known report of HPV prevalence in OSSN in a South African cohort. The HPV prevalence is high and in keeping with other low-middle income countries. Majority of patients with confirmed OSSN are HIV positive, and a lower CD4 count may be linked to a more invasive form of OSSN. HPV subtypes that were most common in this cohort include 11,16 and 18, with many samples having more than one positive genotype.

## Data Availability

Anonymized data sets generated are available from the corresponding author on reasonable request and with permission from Stellenbosch University.

## Abbreviations

HPV: Human papillomavirus
OSSN: Ocular surface squamous neoplasia
HIV: Human immunodeficiency virus
NHLS: National health laboratory service
VL: Viral load
FFPE: Formalin-fixed paraffin embedded tissue
PCR: Polymerase chain reaction

## References

[1] Kiire CA, Srinivasan S, Karp CL. Ocular surface squamous neoplasia. Int Ophthalmol Clin. 2010. 10.1097/IIO.0b013e3181e246e5. 20611016 10.1097/IIO.0b013e3181e246e5

[2] Basti S, Macsai MS. Ocular surface squamous neoplasia: a review. Cornea. 2003. 10.1097/00003226-200310000-00015. 14508267 10.1097/00003226-200310000-00015

[3] Gichuhi S, Ohnuma SI, Sagoo MS, Burton MJ. Pathophysiology of ocular surface squamous neoplasia. Exp Eye Res. 2014. 10.1016/j.exer.2014.10.015. 25447808 10.1016/j.exer.2014.10.015 PMC4726664

[4] Bowling B. Ocular tumours. In: Boling B, editor. Kanski's clinical ophthalmology: a systematicapproach. 8th ed. Elsevier; 2016. p. 476–7.

[5] Chalkia AK, Bontzos G, Spandidos DA, Detorakis ET. Human papillomavirus infection and ocular surface disease (review). Int J Oncol. 2019. 10.3892/ijo.2019.4755. 30896784 10.3892/ijo.2019.4755 PMC6438422

[6] Ateenyi-Agaba C. Conjunctival squamous-cell carcinoma associated with HIV infection in Kampala. Uganda Lancet. 1995. 10.1016/S0140-6736(95)90870-6. 7885126 10.1016/S0140-6736(95)90870-6

[7] Simbiri KO, Murakami M, Feldman M, et al. Multiple oncogenic viruses identified in Ocular surface squamous neoplasia in HIV-1 patients. Infect Agent Cancer. 2010. 10.1186/1750-9378-5-6. 20346104 10.1186/1750-9378-5-6 PMC2859758

[8] Yu JJ, Fu P, Pink JJ, et al. HPV infection and EGFR activation/alteration in HIV-infected East African patients with conjunctival carcinoma. PLoS One. 2010. 10.1371/journal.pone.0010477. 20498858 10.1371/journal.pone.0010477 PMC2871792

[9] Kim BH, Kim MK, Wee WR, Oh JY. Clinical and pathological characteristics of ocular surface squamous neoplasia in an Asian population. Graefe’s Arch Clin Exp Ophthalmol. 2013. 10.1007/s00417-013-2450-0. 10.1007/s00417-013-2450-0 24006080

[10] Lee GA, Hirst LW. Retrospective study of ocular surface squamous neoplasia. Aust N Z J Ophthalmol. 1997. 10.1111/j.1442-9071.1997.tb01514.x. 9395829 10.1111/j.1442-9071.1997.tb01514.x

[11] Kabra RC, Khaitan IA. Comparative analysis of clinical factors associated with ocular surface squamous neoplasia in HIV infected and non HIV patients. J Clin Diagn Res. 2015. 10.7860/JCDR/2015/13236.5932. 26155504 10.7860/JCDR/2015/13236.5932 PMC4484096

[12] Maudgil A, Patel T, Rundle P, Rennie IG, Mudhar HS. Ocular surface squamous neoplasia: analysis of 78 cases from a UK ocular oncology centre. Br J Ophthalmol. 2013. 10.1136/bjophthalmol-2013-303338. 24064943 10.1136/bjophthalmol-2013-303338

[13] Porges Y, Groisman GM. Prevalence of HIV with conjunctival squamous cell neoplasia in an African provincial hospital. Cornea. 2003. 10.1097/00003226-200301000-00001. 12502938 10.1097/00003226-200301000-00001

[14] Muñoz N, Bosch FX, de Sanjosé S, et al. Epidemiologic Classification of Human Papillomavirus Types Associated with Cervical Cancer. N Engl J Med. 2003. 10.1056/nejmoa021641. 12571259 10.1056/nejmoa021641

[15] Guthoff R, Marx A, Stroebel P. No evidence for a pathogenic role of human papillomavirus infection in ocular surface squamous neoplasia in Germany. Curr Eye Res. 2009. 10.1080/02713680903007162. 19899994 10.1080/02713680903007162

[16] Hӧllhumer R, Michelow P, Williams S. Demographics, clinical presentation and risk factors of ocular surface squamous neoplasia at a tertiary hospital, South Africa. Eye (Basingstoke). 2023. 10.1038/s41433-023-02565-1. 10.1038/s41433-023-02565-1 PMC10686408 37258660

[17] Gichuhi S, Sagoo MS, Weiss HA, Burton MJ. Epidemiology of ocular surface squamous neoplasia in Africa. Tropical Med Int Health. 2013. 10.1111/tmi.12203. 10.1111/tmi.12203 PMC4440345 24237784

[18] CDC (2021) HPV Vaccination: What Everyone Should Know | CDC. (Centros para el Control y la Prevención de Enfermedades).

[19] Bruni L, Saura-Lázaro A, Montoliu A, et al. HPV vaccination introduction worldwide and WHO and UNICEF estimates of national HPV immunization coverage 2010–2019. Prev Med (Baltim). 2021. 10.1016/j.ypmed.2020.106399. 10.1016/j.ypmed.2020.106399 33388322

[20] Delany-Moretlwe S, Kelley KF, James S, et al. Human papillomavirus vaccine introduction in South Africa: implementation lessons from an evaluation of the national school-based vaccination campaign. Glob Health Sci Pract. 2018. 10.9745/GHSP-D-18-00090. 30143561 10.9745/GHSP-D-18-00090 PMC6172125

